# Improved host-plant resistance to *Phytophthora* rot and powdery mildew in soybean (*Glycine max* (L.) Merr.)

**DOI:** 10.1038/s41598-020-70702-x

**Published:** 2020-08-18

**Authors:** Jegadeesan Ramalingam, Ganesh Alagarasan, Palanisamy Savitha, Kelsey Lydia, Govindan Pothiraj, Eswaramoorthy Vijayakumar, Rajaprakasam Sudhagar, Amar Singh, Kumari Vedna, Chockalingam Vanniarajan

**Affiliations:** 1 Centre for Plant Molecular Biology and Biotechnology, Tamil Nadu Agricultural University, Coimbatore, India; 2 Department of Biotechnology, Agricultural College and Research Institute, Tamil Nadu Agricultural University, Madurai, India; 3 Department of Plant Breeding and Genetics, Agricultural College and Research Institute, Tamil Nadu Agricultural University, Madurai, India; 4 Centre for Plant Breeding and Genetics, Department of Pulses, Tamil Nadu Agricultural University, Coimbatore, India; 5 Department of Plant Pathology, Chaudhary Sarwan Kumar Himachal Pradesh Krishi Vishvavidyalaya, Palampur, India; 6 Department of Plant Breeding and Genetics, Chaudhary Sarwan Kumar Himachal Pradesh Krishi Vishvavidyalaya, Palampur, India

**Keywords:** Plant biotechnology, Plant breeding

## Abstract

Soybean is an important oilseed cum vegetable crop, susceptible to various biotic stresses which is attributed to recent decline in crop productivity. The emergence of virulent biotypes/strains of different plant pathogens necessitates the development of new crop varieties with enhanced host resistance mechanisms. Pyramiding of multiple disease-resistant genes is one of the strategies employed to develop durable disease-resistant cultivars to the prevailing and emerging biotypes of pathogens. The present study, reports the successful introgression of two major R-genes, including *Rps2* (*Phytophthora* rot resistance), *Rmd-c* (complete-powdery mildew resistance) and effective nodulating gene (*rj2*) through functional Marker-Assisted Backcross Breeding (MABB) in the genetic background of well-adapted and high yielding soybean varieties, CO 3 and JS 335. We have identified several promising introgressed lines with enhanced resistance to *Phytophthora* rot and powdery mildew. The improved soybean lines have exhibited medium to high level of resistance against powdery mildew and *Phytophthora* rot as well as displayed effective nodulation capacity. Our study has proven the generation of resistant genotypes to realize the potential of MABB for achieving host plant resistance in soybean. The improved lines developed can greatly assist the soybean breeding programs in India and other soybean growing countries for evolving disease-resistant varieties.

## Introduction

*Phytophthora* root and stem rot and powdery mildew are the widespread diseases of soybean crop affecting all major plant parts including seeds, stems, leaves, and roots. Many of the high yielding soybean mega-varieties are highly susceptible to both diseases^1–3^. Infections by these pathogens cause severe tissue collapse in host plant that negatively impacts plant growth and productivity. Many of the chemical, cultural and biological methods suggested are practically inadequate to combat these diseases successfully^1,4,5^. However, a sustainable approach for combating pathogenesis is to introgress the disease resistant (R-genes) and QTL from the identified soybean lines in to cultivars^5^. Although many research groups have successfully introgressed R-genes into an elite variety^6,7^ through marker-assisted breeding, there are very few reports of utilizing marker-assisted backcross breeding (MABB) method. Successful MABB was reported in soybean for mobilizing the null allele of β‐conglycinin α‐subunit^8^ and Kunitz trypsin inhibitor^9^. Following this example, it will be valuable to introgress the R-gene(s) through MABB to achieve high recurrent parent genome recovery (RPG) while eliminating the linkage-drag effect on target chromosome(s).

Upon pathogen invasion, resistance to development of *Phytophthora* rot and powdery mildew diseases are conferred by *Rps* and *Rmd* genes respectively and the resistance attributed being effector-triggered immunity (ETI) in plants^10,11^. So far 30 *Rps* genes (*Rps1a, b, c, d* and *k, Rps2, RpsWY,* etc.) and 3 alleles at the *Rmd* locus (*Rmd-c, Rmd,* and *rmd*) have been identified and fine-mapped in the resistant soybean cultivars^12^. Many studies have mapped the *Rmd* gene between *Rj2* (non-nodulating) and *Rps2* genes on LG J (linkage group J)^10,13^. CNS is the source of resistance to powdery mildew disease and controlled by a single allele (*Rmd-c*), was shown to offer resistance throughout the entire life cycle of plant^14^. Another gene, *RpsUN2* different from *Rps2* was mapped in this region and is located approximately 0.8 cM downstream of *Rj2*, and the genetic distance between *RpsUN2* and *Rps2* is between 3.0 and 3.4 cM^15^. The list of popular *Rps* genes and near-isogenic lines (NILs) carrying *Phytophthora* root and stem rot and powdery mildew resistance genes and the source of resistance were already reported^16^. *Rps2* is a broad-spectrum resistance gene first reported in soybean line D54-2437, derived from the genotype namely CNS. *Rps2* provides resistance to at least 18 of the 27 reported races of *P. sojae*^17^. This gene provides partial resistance, which is a form of incomplete resistance to *Phytophthora* and effective against all races of the pathogen with a reduced level of root colonization^11^. The location of two essential genes (*Rps2* and *Rmd*) in a relatively small region, and its linkage to several markers and the availability of isolines and segregating population make this portion of linkage group J, a prime candidate for developing NILs population in soybean. *Rmd, Rj2, and Rps2* genes map in 3.8 cM region of the linkage group of J^10^. Large groups of resistance gene analogs (RGA) were mapped in this region^18^. They also mapped RGA BAC (bacterial artificial chromosomes) with in 1.8 cM region between these genes. A physical map of soybean cv. Williams 82 and cv. Faribault was initially developed using the BAC library^19^. Later, a BAC contig was developed in this region using different RGA specific primers and fingerprinting^20^. On the opposite ends of a core BAC in the contig Gm_Isb001_091_F11 (BAC 91F11), two TIR/NBD/LRR (Toll-interleukin receptors/nucleotide binding domain/leucine-rich repeats) cDNAs were also mapped^20^. The complete sequence of BAC 91F11 from cultivar Williams 82 is now available and it harbors 16 different R-gene sequences, including four R-genes from two potentially novel classes of disease resistance genes^20^. Interestingly, Wu et al.^21^ generated a BAC contig from the cultivar viz. Forrest and Williams 82 to create an extensive, durable disease–resistance gene clusters, including *Rmd, Rj2,* and *Rps2* genes. Around fifteen different R-genes were identified in this BAC region. We have developed several PCR-based markers using end sequence of BAC 91F11 and tested with near isogenic lines carrying *Rmd-c Rj2,* and *Rps2* in the background of Williams. The functional markers derived from polymorphic sites within gene sequences affecting phenotypic variation is more efficient for gene identification and selection^22^. In our study, we have used some of the functional markers linked to *Rmd-c Rj2*, and along with other molecular tags like K375 and Satt_144. Both K375 and Satt_144 are anchor markers located with RGA 1 region in J linkage map18, 20 which harbors *Rmd-c, Rj2* and *Rps 2* genes.

In India, soybean is cultivated as a rainfed crop and the incidence of *Phytophthora* rot disease was mostly noticed in the rainfed ecosystem after heavy rainfall, causing substantial yield losses. Powdery mildew is more prevalent in the northern part of India. Considering the economic importance of these diseases, we have planned to introgress the *Rps2* along with the *Rmd-c* genes to develop durable resistance to both diseases. The non-availability of diverse disease-resistant soybean lines or varieties further limits the disease breeding program in India. Considering the needs of farmers and growing demand for resistant cultivars, we preferred using locally well-adapted soybean lines as the base genetic material for introgression. Development of well-adapted *Phytophthora* rot and powdery mildew resistant cultivars as well as with effective nodulation ability may offer a source material for the soybean breeding program in India and elsewhere.

## Materials and methods

### Plant materials

**Figure 1:**
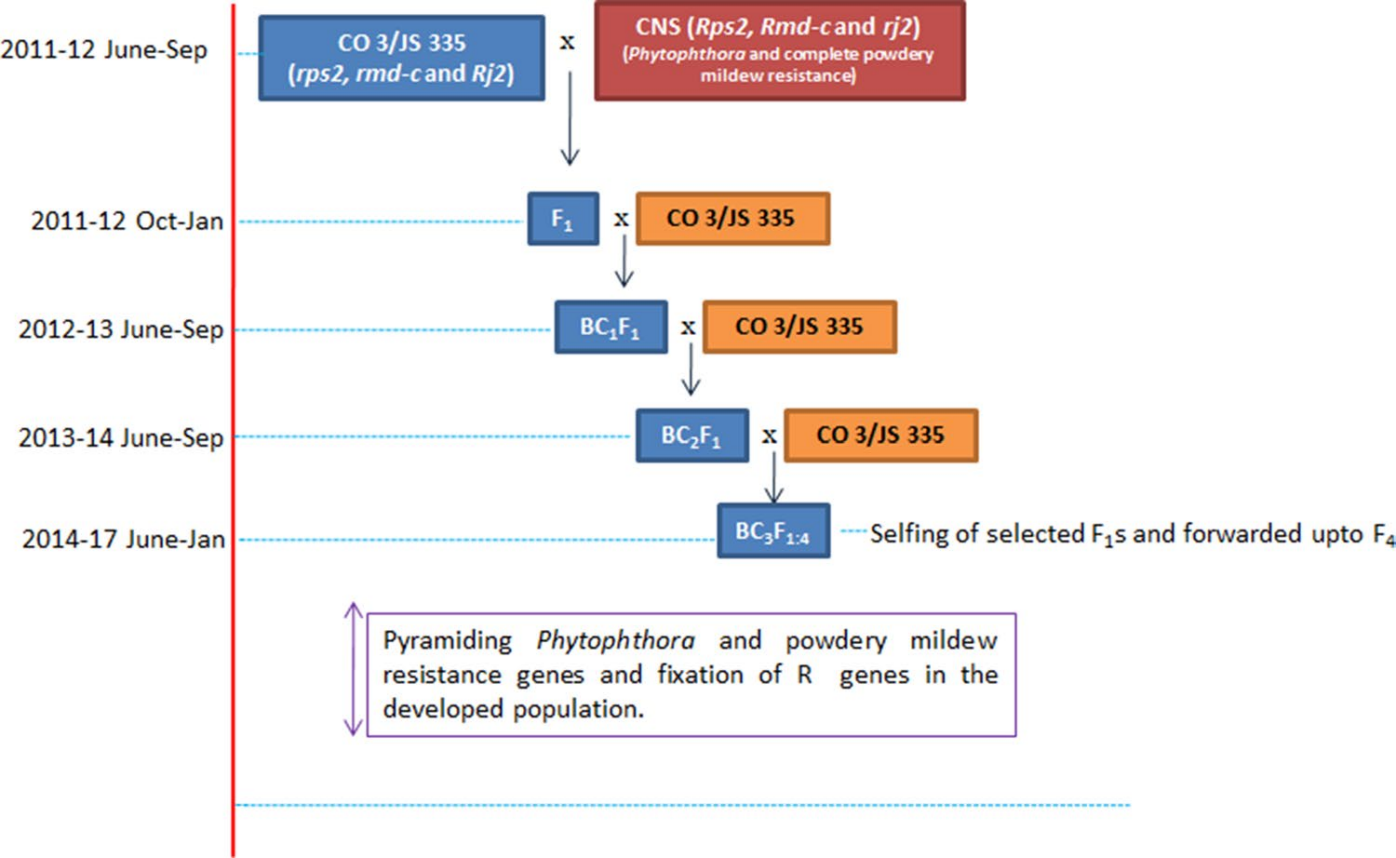
Schematic representation of integration of *Rps2, Rmd-c* and *rj2* genes into popular soybean varieties, CO 3 and JS 335. Selected individual plants with high recurrent parent genome content and exhibiting high level of resistance were used for further crossing, in both the independent crosses made.

We have used the high yielding nodulating genotypes viz., CO 3 (cultivated in South India) and JS 335 (grown in North India) as the recurrent parents in this crossing program. The collected donor line CNS from the World Vegetable Centre (AVRDC), Taiwan was used as a donor parent for *Phytophthora* and complete powdery mildew resistance (*Rps2* + *Rmd-c*). The donor line, CNS has an ineffective nodulation gene (*Rj2*) in the J linkage group. The recurrent parents carry effective nodulation gene, *rj2*. A brief crossing program layout and genotype of recipient and donor lines is given in Fig. 1. The high yielding elite varieties, and powdery mildew, *Phytophthora* rot resistant donor lines were raised by staggered sowing method at an interval of two-weeks to achieve programmed pollination. All the cultural operations were carried out as per the soybean crop cultivation guide (https://agritech.tnau.ac.in). The crossing between parents was made in the early morning hours but not later than 8 AM, Indian standard time (IST). The entire field experiments were conducted in sandy loam and clay loam soils at Tamil Nadu Agricultural University farms, Coimbatore from 2011 to 2018.

### Foreground and background selection for *Phytophthora* and powdery mildew resistance

Young leaves were used for genomic DNA extraction using the modified cetyltrimethylammonium bromide (CTAB) protocol^23^. Quality and quantity assessment of DNA was performed on 0.8% agarose gels. DNA samples were diluted to a concentration of 50 ng/µL with ddH_2_O and stored at − 20 °C for further use. Polymerase Chain Reaction (PCR) based molecular markers linked to *Rps*2, *Rj2* and *Rmd-c* genes (foreground selection) as well as SSR markers (background selection) were used to screen the parents and progenies in each backcrossed/selfed generation. The detailed information on molecular markers is provided in Table 1. These markers were developed based on BAC end sequences of BAC 91 F11. The primers were developed and confirmed in the Williams near isogenic lines, L 76-1988 (*Rps2, Rj2,* and *Rmd-c*: Resistant) and L 82-2024 (*rps2, rj2,* and *rmd-c*: Susceptible) and its segregating population (Unpublished data). The PCR reaction conditions were: 2 min initial denaturation at 94 °C followed by 35 cycles as follows: 91 °C for 1 min, 55 °C for 30 s, and 72 °C for 1 min.

**Table 1:** Markers used in foreground analysis of *Phytophthora* rot and powdery mildew resistance and effective nodulating genes.

Marker	Primer sequence (5′-3′)*	Enzyme	References	Parental polymorphism
91F11L	**F**-GAAAGTTCATAAAGTCGTA **R**-TATGACTAACAGGAACTGC	*TaqI*	^39^	Polymorphic
Sat144	**F**-GCGCCCTCTTCATTTCCCTTGGTT **R**-GCGCCCAATATCTTTGGGATAAAT	–	^40^	Monomorphic
91F11L-R2	**F**-AAG ATA AGA TGG AGA AGC TGC **R**-AAG ATA AGA TGG AGA AGC TGC	*Hind III*	^20^	Monomorphic
91F11L-R10	**F**-GGA TTA TCC AGT TGG TCT AGG **R**-GCT CGC GCT TAT CAA CAT CG	*Hind III*	^20^	Monomorphic
91F11L-R14	**F**-TAG TTA TGG AGA AGC AAT GAT **R**-ATT GCT CGT GCC TGT CTG	*Hind III*	^20^	Monomorphic
89L6R-R 89L6R-1B2	**F**-GAA CTT CAG TAC TTG AC **R**-TCG TTG TTTATCCATTCCCG	–	^20^	Monomorphic
89L6R-R 89L6R-1B	**F**-GAA CTT CAG TAC TTG AC **R**-TGG TTG TTT ATC CAT TCC CA	–	^20^	Monomorphic
K375	**F**-ACC ATT AGG ACT GAG TTT G **R**-GCT TGA ATA GCG ATC CTT C	–	^20^	Monomorphic

*F, forward; **R**, reverse.

Also, we have used soybean microsatellite (SATT) markers providing genome-wide coverage for parental polymorphism survey between the donor and recurrent parents. Only the polymorphic markers were used for background selection. The different set of SSR markers were synthesized from Bio serves Chemicals Pvt Ltd., Bangalore. In total, 125 microsatellite loci that span the whole soybean genome were screened for the parental polymorphism. The SSR markers that showed polymorphism between recurrent parent (CO 3 and JS 335) and donor (CNS) were used to screen the foreground of selected progeny in F_1_, BC_1_, BC_2_ until the BC_3_ generation.

### Evaluation of powdery mildew resistance

A survey was conducted on naturally infected (powdery mildew) plants of soybean, and based on the conidiophore morphology, the pathogen was identified as *Microsphaera diffusa.* The density of the conidial population was approximately of 750 spores/cm^2^. The spore suspension with a concentration of 1 × 10^4^ ml^−1^ was prepared for artificial inoculation^24^. A total number of 25 seeds in three replicates for each genotype viz., CO 3, JS 335, CNS and introgressed lines were raised in a greenhouse (25–27 °C, 14 h light, 70% relative humidity) in autoclaved sand benches. One week after planting, *Microsphaera diffusa* was inoculated with a fresh conidial suspension (1 × 10^4^ ml^−1^) on the fully expanded unifoliate leaves of the plant^23^. Plants were scored for diseases according to a 0–5 grade scale^25^, as follows: 0 = no leaf symptoms, 1 = 10% of the leaf surface with symptoms, 2 = 11–25% of the leaf surface with symptoms, 3 = 26–50% of the leaf surface with symptoms, 4 = 51–75% of the leaf surface with symptoms, 5 = more than 75% of the leaf surface with symptoms. In the present study, plants scored as grades 0–1 were considered highly resistant (HR), 1–2 as moderate resistant (MR), 2–3 as moderate susceptible (MS) and 4–5 taken as highly susceptible (HS). The improved lines and parents were also grown in hot spot region of Palampur, Himachal Pradesh, India to confirm the field reaction.

Detached Leaf Assay (DLA) was also conducted to confirm the resistance reaction^24^. A trifoliate leaf was placed in a plastic petri dishes containing a thin layered moist blotting paper. The leaves were spray inoculated using freshly prepared spore suspension (1 × 10^4^ ml^−1^) and the inoculated plates were incubated for 24 h at 24 ± 1 °C. Subsequently, the plates were incubated for two weeks under fluorescence light at 24 ± 2 °C and evaluated for disease reaction as powdery patches/lesions in the inoculated leaves.

### *Phytophthora* rot resistance screening

A total of ten seeds per line in three replicates were planted in the greenhouse in sterilized clay pots using a sterilized soil: perlite: sand media. The susceptible parents, CO 3 and JS 335 and resistant donor line CNS were used as positive and negative controls. A culture of *Phytophthora sojae* was isolated from the infected soybean plants and cultured in oatmeal agar media (Table S1). The purity of *Phytophthora* culture was checked by its growth on V8 juice agar media with a piece of fungus (0.5 cm^2^) transferred from oatmeal agar. *Phytophthora sojae* inoculum was prepared by growing on oatmeal agar in petri dishes at room temperature under darkness for 10 days. Just before inoculation, petri dish with culture was inverted and the agar and culture transferred on to a sterile paper. The agar medium was carefully scraped away leaving intact the surface layer of mycelia. The mycelium layer was then cut into small pieces, approx. 0.5 mm by 1.5 mm. After five to six days of planting and before the opening of the unifoliate leaf, uniform sized seedlings were selected for inoculation. A slit approximately 4 mm in length was made 0.5 cm below the hypocotyl hook with a sterile knife, and a piece of fungal mycelia was introduced into the slit^26^. After inoculation, the seedlings were kept in humid chamber for 24 h. Following inoculation, plants were brought out onto the greenhouse bench.

Scoring was carried out four days after inoculation. Susceptible phenotype showed withered, and blackened cotyledons above the slit and a slightly shrunken hypocotyl, and stem still green just at the soil level. Healthy, resistant plants formed scar tissue around the incision. Testing was repeated on lines that were scored homozygous for resistance or susceptibility. The donor lines were tested under greenhouse in Iowa State University, USA. The homozygous improved lines and parents were tested and confirmed under field conditions in India.

### Screening for nodulation efficiency

A pure culture of *Rhizobium japonicum* soybean strain (USDA 122) was received from the Department of Agricultural Microbiology, Tamil Nadu Agricultural University, Coimbatore and used for the preparation of inoculant in yeast mannitol liquid broth culture (Table S2). Four-day-old *Rhizobium* cultures (50 ml) were centrifuged and the cell pellet was suspended in a volume of plant nutrient solution lacking any nitrogen source (PNS-N) and adjusted to a final absorbance of 0.800 at 650 nm. Dry seeds of parents and improved lines were inoculated with the *Rhizobium* inoculum by slowly pipetting 1 ml rhizobia-PNS-N solution onto seed surface. The seeds were planted in the pots containing autoclaved vermiculate medium and wetted with double distilled water. PNS-N was administered once a week in place of double distilled water beginning seven days after planting at a rate of 100 ml per pot.

The plants were scored for nitrogen deficiency after 21 days of planting. Green plants were scored as effective nodulator *(rj2*/*rj2*), and yellow plants were recorded as ineffective nodulator (*Rj2*/*Rj2*). To confirm the phenotypic scoring, plants were randomly lifted to inspect roots for nodule formation. Nodules were found on primary and secondary roots proximal to the hypocotyl region in green plants. The nodules were examined morphologically and anatomically by sectioning.

### Agronomic performance

Twelve improved lines (80–100 plants per line) in BC_3_F_4_ of CO 3 × CNS and JS 335 × CNS cross combinations were evaluated along with their parents (CO 3, JS 335, and CNS) in a randomized block design with three replications. Biometrical observations including plant height, days to 50% flowering, days to maturity, number of primary branches, number of clusters per plant, number of pods per plant, number of seeds per pod, 100 seed weight and seed yield per plant were recorded on randomly selected twenty five plants in each entry in each replication. The data was subjected to statistical analysis with STAR nebula 2.0.1 statistical package developed by International Rice Research Institute (IRRI).

## Results

### Parental polymorphism analysis

**Figure 2:**
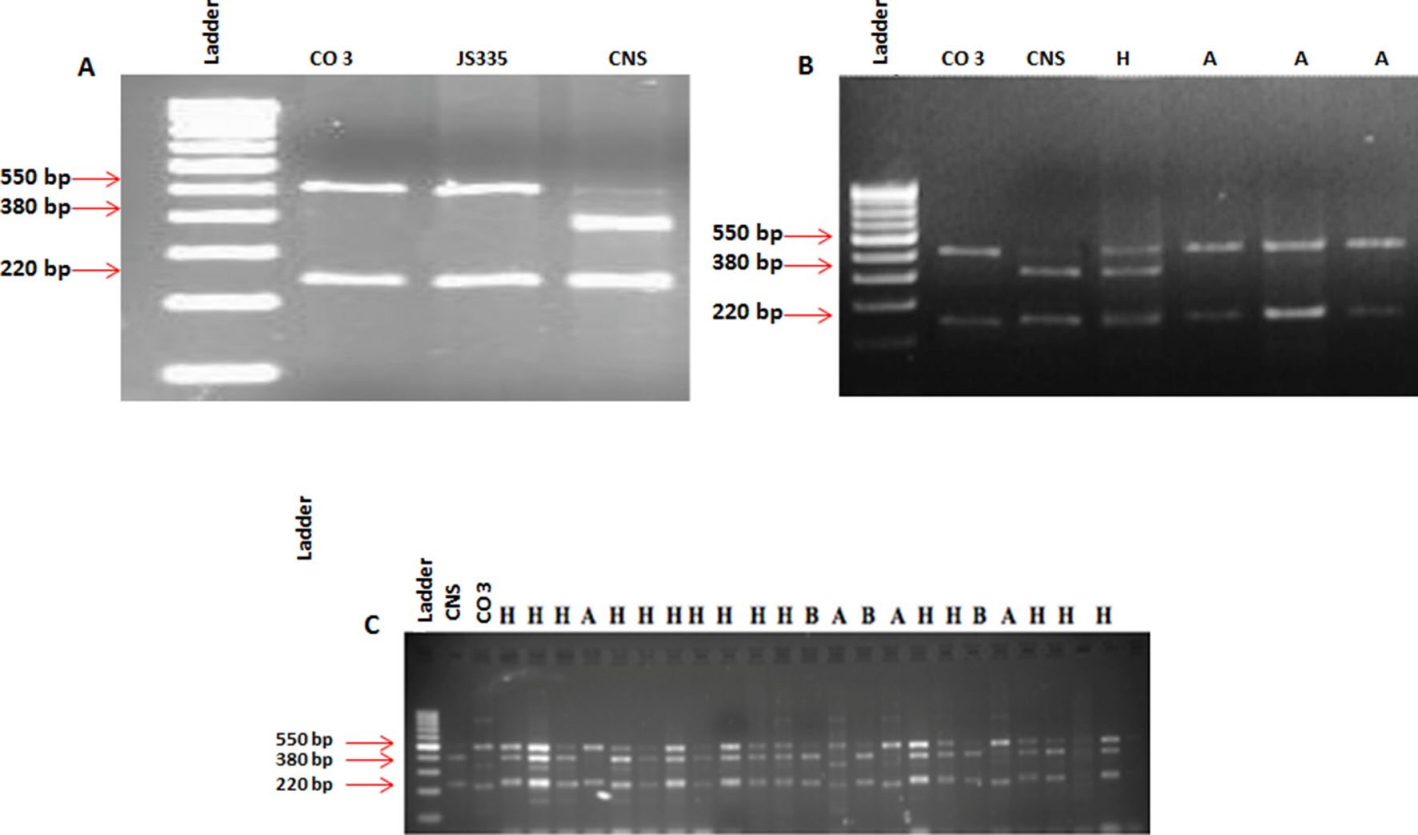
Foreground analysis using 93F11L marker. (**A**) Polymorphism survey between parents, (**B**) fixing of hybrids in F_1_ generation and BC_3_F_2_, (**C**) screening of plants. H- plants carrying alleles from both parents, A- allele from recurrent parent (CO 3) and B- allele from donor parent (CNS).

PCR-based markers developed based on BAC end sequences, and linked SSR markers with *Rps2/Rmd-c/rj2* gene in the J linkage group were tested for polymorphism. Parental polymorphism survey through molecular markers revealed the distinct banding pattern of the genes/alleles to be introgressed into susceptible recipient parent, i.e., CO 3 and JS 335 from resistant donor parent, i.e., CNS (91F11L: *Rps2* + *Rj2* + *Rmd-c*) (Fig. 2). None of the other markers showed clear distinct difference in banding pattern between the parents (Fig. S1). 91F11L which was developed from BAC end sequence showed complete co-segregation with phenotypes of resistant and susceptible near isogenic lines (L 1988 and L 2021) and differed for *Rps2*, *Rmd-c* and *rj2*. These NILs differ for these three genes in the background of Williams. A mapping population was also developed using these NILs and the F_3_ and F_4_ plants were genotyped and phenotyped. A perfect genotype with phenotype relationship was obtained (unpublished data) with BAC 91F11L functional marker. In the bioassays conducted against powdery mildew, the donor parent (CNS) showed a higher level of resistance, while the recipient parents (CO 3 and JS 335) were susceptible to powdery mildew. Similarly, the donor parent, CNS showed high resistance to *Phytophthora sojae* and ineffective nodulation.

### Pyramiding of resistance genes

The high yielding Indian soybean varieties, CO 3 and JS 335, which are susceptible to *Phytophthora* and powdery mildew diseases were introgressed with *Rps2*, *rj2*, and *Rmd-c* through MABB. Two independent conventional backcross methods were followed to transfer R-genes in CO 3 and JS 335. In the first phase, the CO 3 and JS 335 were individually crossed with both the donor parents and each of the F_1_s was backcrossed to the respective recipient parent. The developed population was maintained and advanced till BC_3_F_4_ generation. At each backcross cycle, the derived progenies were screened through functional markers to confirm the presence of desired R-genes and effective nodulation gene, *rj2*. The stringent phenotypic selection was employed to select the progenies that are phenotypically similar to the recipient parents, along with target resistance and nodulation genes.

### Foreground and background selection

The genotypic selection was done in parents, F_1_, BC_1_F_1,_ BC_2_F_1_ and till BC_3_F_1_ generation using functional marker 91 F11L linked to *Phytophthora* and powdery mildew resistance and effective nodulation genes. For the selection of *Phytophthora* rot and powdery mildew resistant traits and effective nodulation in the segregating population, the effective foreground marker should be identified which is polymorphic for recurrent and donor parents. It was found that the functional marker 91F11L (*Rps2, Rj2* and *Rmd-c*) was found to be polymorphic between resistant and susceptible allele. Other markers showed monomorphism among the parents. This functional marker 91F11L was used in subsequent screening of the segregating population in MABB for foreground selection. The hybridity of the F_1_ plants was confirmed using this marker between the parental lines (Fig. 2). The F_1_ plants were analyzed for the presence of both dominant and recessive alleles (heterozygous band). The banding pattern of resistant and susceptible parents was used as a reference for the identification of true hybrids. From the cross derived F_1_ plants, individuals showing single band (either dominant allele or recessive allele) were rejected as it indicates selfing. A total of 45 plants from CO 3 × CNS, and 15 plants from JS 335 × CNS were genotyped. Four plants in CO 3 × CNS and five plants in JS 335 × CNS, were identified as true hybrids. The identified hybrids were selfed and backcrossed to develop the near isogenic lines (NILs).

Background analysis was performed in the generation BC_1_F_1_, BC_2_F_1_ and BC_3_F_1_. Of the 125 markers surveyed for parental polymorphism, 39 markers in CO 3 × CNS and JS 335 × CNS exhibited clear polymorphic bands (Fig. S2, Table S3) The extent of recurrent parent genome percentage was gradually increased from BC_1_F_1_ to BC_3_F_1_ (Table 2, Fig S3). Although theoretically we didn’t achieve the expected recurrent parent genome level in the BC_3_F_1_ generation, the developed lines had a similar morphological appearance to the recurrent parent. A single BC_3_F_1_ plant with high parental genome (89.74%) in each cross was selfed and forwarded up to F_4_. The improved homozygous lines were screened for disease reactions, nodulation efficiency and agronomic performance.

**Table 2:** Analysis of recurrent genome contribution in selected backcross lines of both CO 3 × CNS and JS 335 ×  CNS crosses.

Cross	Plant number	No. of polymorphic markers (n)	No. of homozygous markers (x)	No. of heterozygous markers (y)	Recurrent genome contribution (G) %
**BC**_**1**_**F**_**1**_					
CO 3 × CNS	a2-1-1	39	30	9	76.92
	a2-1-2	39	33	6	84.61
	a2-1-3	39	33	6	84.61
JS 335 × CNS	c2-1-1	39	31	8	79.48
	c2-1-2	39	29	10	74.35
	c2-1-3	39	32	7	82.05
**BC**_**2**_**F**_**1**_					
CO 3 × CNS	a3-1-1	39	32	7	82.05
	a3-1-2	39	33	6	84.61
	a3-1-3	39	34	5	87.17
JS 335 × CNS	c3-1-1	39	31	8	79.48
	c3-1-2	39	33	6	84.61
	c3-1-3	39	33	6	84.61
**BC**_**3**_**F**_**1**_					
CO 3 × CNS	a4-1-1	39	33	6	84.61
	a4-1-2	39	33	6	84.61
	a4-1-3	39	35	4	89.74
JS 335 × CNS	c4-1-1	39	32	7	82.05
	c4-1-2	39	34	5	87.17
	c4-1-3	39	35	4	89.74

### Disease resistance and nodulation analysis

**Figure 3:**
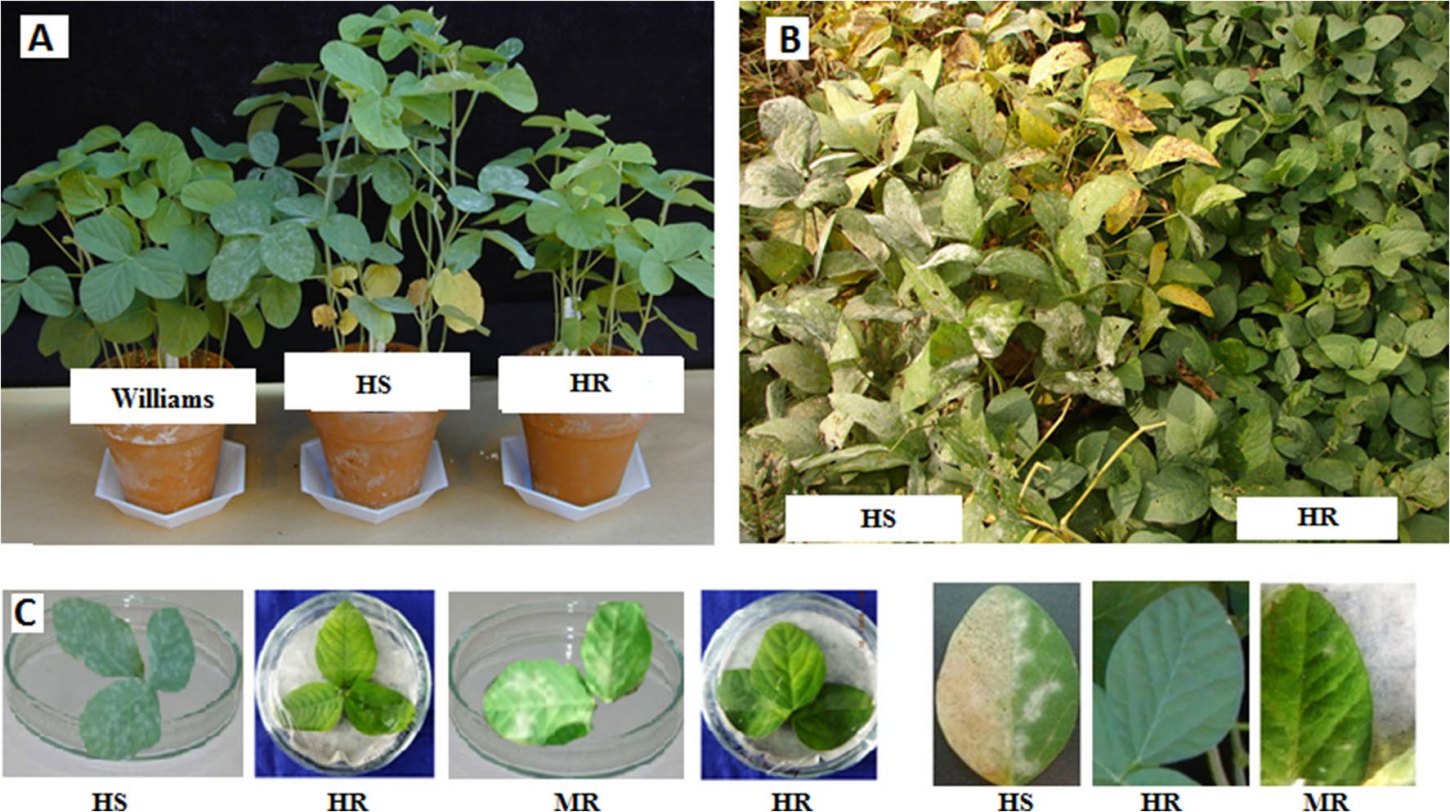
Powdery mildew screening of parents and improved lines. (**A**) Whole plant assay. check (Williams carrying *Rmd* gene), HS (highly-susceptible) genotype (CO 3 carrying *rmd*-c gene) and HR (highly-resistant) genotype (CNS carrying *Rmd*-c). (**B**) Field screening of CO 3 (HS) and improved CO 3 (HR) genotypes against powdery mildew. (**C**) Detached leaf assay. From the left, three leaf view of genotype showing HS (CO 3), HR (CNS), MR (moderately-resistant) and HR (improved CO 3) reaction. Close view of individual leaf showing HS (CO 3), resistant (CNS) and moderately resistant (improved CO 3) reaction.

The plant genotypes were screened for powdery mildew resistance through whole plant assay (WPA) and detached leaf assay (DLA) in the greenhouse condition. In WPA, a total of 25 seedlings in each parent viz., CO 3, JS 335, CNS and improved lines were grown in autoclaved sand bench under greenhouse condition. The improved lines and CNS were resistant while the high yielding varieties CO 3 and JS 335 were highly susceptible to powdery mildew (Fig. 3). After 20 days, most of the improved lines, and CNS showed no symptoms while the high yielding varieties CO 3 and JS 335 showed sparse mycelial growth. Improved CO 3 lines (1 and 4) and Improved JS 335 lines (1 and 3) showed moderate resistance, while all other lines showed high resistance reaction against powdery mildew pathogen (Table 3 and Fig. S4). Through DLA analysis, powdery patches/lesions were observed on the abaxial surface of inoculated leaves (Fig. 3). Improved CO 3 lines showed moderate to high resistant reaction compared to susceptible CO 3. Twelve improved lines and parents were also grown in hotspot area to confirm the resistant reaction against powdery mildew. All the improved lines showed moderate to high resistance reaction (Fig. S5).

**Table 3:** Reaction of improved lines and its parents for powdery mildew and *Phytophthora* rot resistance and nodulation efficiency.

Pyramid/introgression lines with gene combinations	Powdery mildew disease score	Reaction	*Phytophthora* rot reaction	Nodulation efficiency
**CO 3 × CNS (Rps2,rj2 and Rmd-c)**				
Imp CO 3-1	2	MR	R	EN
Imp CO 3-2	0	HR	R	EN
Imp CO 3-3	1	HR	R	EN
Imp CO 3-4	2	MR	R	EN
Imp CO 3-5	1	HR	R	EN
Imp CO 3-6	0	HR	R	EN
**JS 335 × CNS (Rps2,rj2 and Rmd-c)**				
Imp JS 335-1	2	MR	R	EN
Imp JS 335-2	1	HR	R	EN
Imp JS 335-3	2	MR	R	EN
Imp JS 335-4	0	HR	R	EN
Imp JS 335-5	0	HR	R	EN
Imp JS 335-6	0	HR	R	EN
CO 3 (recipient/susceptible parent)	5	HS	S	EN
JS 335 (recipient/susceptible parent)	4	HS	S	EN
CNS (donor/resistant parent)	0	HR	R	IN

Plants were scored for powdery mildew disease incidence, according to the 0–5 grade scale: 0–1 = highly resistant (HR), 1–2 = moderate resistant (MR), 2–3 = moderate susceptible (MS), 4–5 = highly susceptible (HS). Modified hypocotyl puncture method was followed to test the *Phytophthora* reaction. Nodulation efficiency was scored as effective nodulator (EN) and ineffective nodulator (IN)^41^.

**Figure 4:**
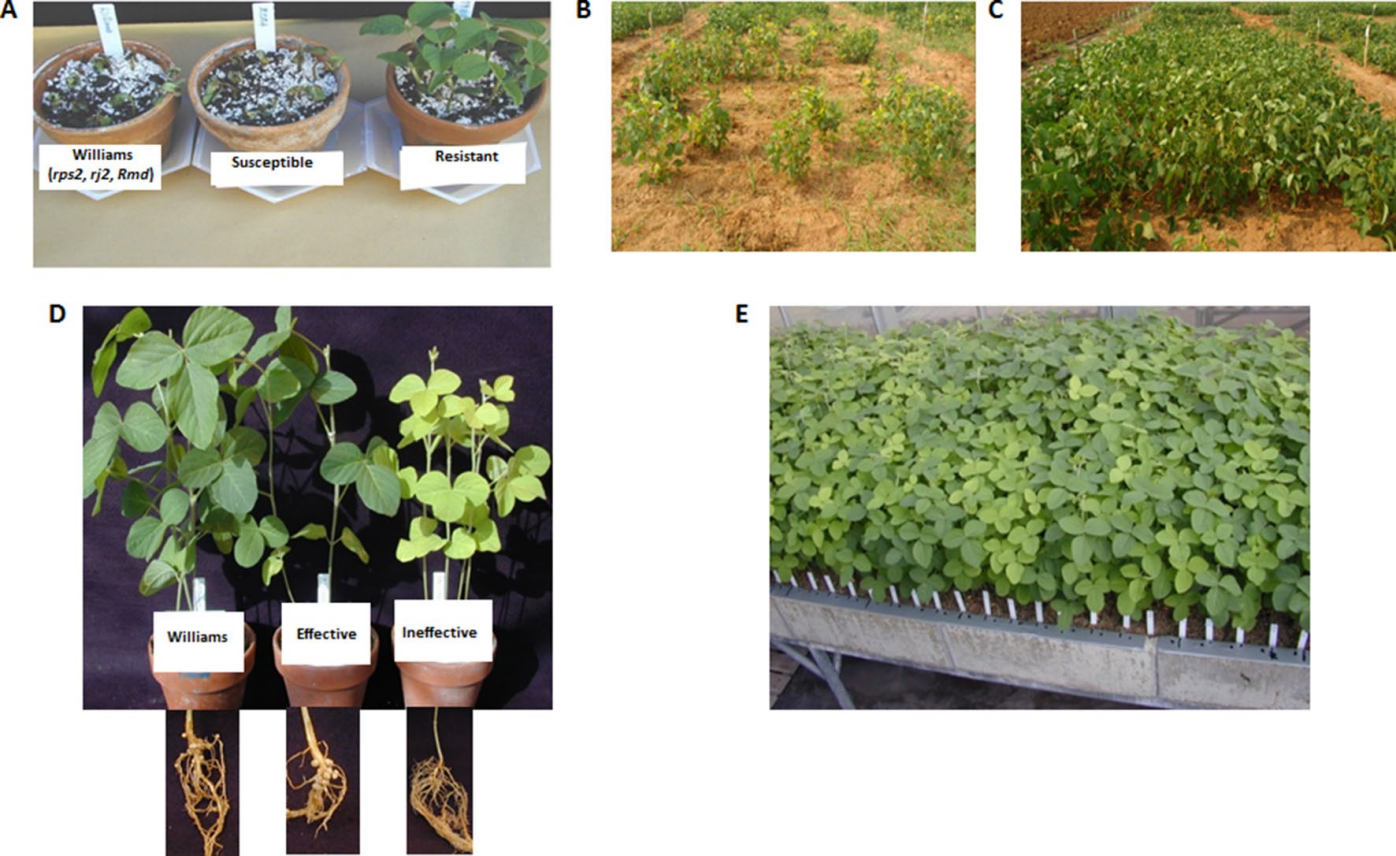
Phenotypic screening for *Phytophthora* and nodulation efficiency (**A**) *Phytophthora* screening for parental lines, susceptible (JS 335), resistant (CNS) and Check (Williams carrying *rps2* and *rj2* genes) (**B**) and (**C**) field screening for resistance against *Phytophthora* (**B**) susceptible parents (**C**) improved back cross lines (**D**) nodulation screening for parental lines, effective nodulation (JS 335), ineffective nodulation (CNS) and check (Williams) (**E**) BC_3_F_2_ lines segregating for effective (green coloured leaves) and ineffective (yellow coloured leaves) nodulation genes.

The improved lines were also screened for resistance against *Phytophthora* rot along with their parents. All the improved lines and donor line carrying *Rmd-c* loci showed resistance against *Phytophthora sojae* pathogen infection (Fig. S4). Whereas, the parents, CO 3 and JS 335 displayed a highly susceptible reaction. These improved lines were also raised in the field condition along with susceptible parents as control check. All the twelve improved lines showed resistant reaction against *Phytophthora sojae* (Table 3). These improved lines along with their parents were also screened for nodulation efficiency. The improved lines harboring *rj2* gene were showing maximum greenish leaves and the roots had maximum number of effective nodules and the plants were looking apparently healthy. Random sample of green plants were uprooted to visualize the nodule development. The nodules were well developed and pink colored upon cross-sectioning. The resistant donor, CNS (*Rj2*) had more yellowish leaves and roots with ineffective nodules. The yellowish seedlings either had no nodules, or infrequently some erupting spot was found on the root epidermis suggesting the rudimentary stage of nodule formation which though failed to develop in to a full nodule subsequently. The phenotypic screening under greenhouse condition for parents and segregating lines in BC_3_F_2_ generation is provided in Fig. 4 and Fig. S4.

### Agronomic performance of improved lines

Improved lines possessing *Rps2*, *rj2* and *Rmd-c* genes in the homozygous state with similar phenotypic appearance to recurrent parents were evaluated for agronomic performance along with their parents in replicated field trials (Table 4, Table S4, Fig. S6). Most of the improved lines with the above gene combinations were significantly superior to their parents for the agronomic traits investigated (Table 4). Five lines showed a significant reduction in days to maturity. Most of the lines had a high number of pods per plant, but only two possessed a high number of seeds per pod compared to their parents. The single plant yield ranged from 19.70 to 40.31 g. Single plant yield was significantly higher in seven pyramided lines (two in CO 3 × CNS and five in JS 335 × CNS). Three lines (plant #3 and 5 in CO 3 × CNS and plant #4 in JS 335 × CNS) were promising as they possess higher single plant yield, more pod clusters, and pods per plant than their parents.

**Table 4:** Agronomic performance of the pyramided lines in comparison to their parents at BC_3_F_4_ generation.

Cross	Plant #	Plant height (cm)	Days to 50% flowering	Days to maturity	No. of primary branch	No. of clusters	No. of pods per plant	No. of seeds per pod	100 seed weight (g)	Single plant yield (g)
CO 3 × CNS	1	26.69	39.80	82.25*	6.00	38.25	115.35	2.60	8.75	23.67
	2	42.39	45.15	90.10	7.20	58.40*	133.50*	2.30	10.26	23.95
	3	67.23*	42.80	89.00	6.95	52.10*	169.40*	2.60	10.46	31.75*
	4	24.18	36.25*	83.05*	6.20	40.20	114.10	2.75	8.45	23.63
	5	53.92	41.20	85.70*	8.55*	58.10*	147.75*	2.70	10.21	36.41*
	6	58.75	50.75	88.45	7.90*	58.60*	146.55*	2.30	8.86	26.12
JS 335 × CNS	1	57.40*	48.10	84.60*	6.20*	26.20	103.05*	2.60	12.52	24.20*
	2	63.27*	40.40	85.20	6.35*	35.45*	111.55*	2.65	8.13	22.46*
	3	41.89*	48.05	74.75*	6.33*	40.75*	104.75*	3.00*	9.86	21.28*
	4	75.12*	41.50	87.50	7.50*	61.10*	171.20*	2.30	12.70	39.34*
	5	33.69*	36.10	85.95	5.30	23.30	56.40	2.60	12.59	19.25
	6	52.23*	41.05	87.10	6.40*	39.35*	101.30*	2.95*	12.73	26.90*
CO 3	–	51.53	38.65	91.05	6.30	38.00	110.65	2.70	10.46	23.44
JS 335	–	22.86	34.40	84.80	4.85	20.75	57.45	2.60	12.45	17.49
CNS	–	44.71	35.40	88.45	5.75	28.90	68.70	2.80	10.81	15.98
SED		3.97	1.24	0.99	0.24	3.38	8.91	0.05	0.41	1.61
CD		8.18	2.54	2.04	0.50	6.96	18.36	0.11	0.85	3.32

*Significantly different from recipient parent.

Agglomerative clustering with Euclidean values divided the soybean improved lines and parents into three clusters cophenetic correlation coefficient = 0.716. Cluster one consisted of three parents and four improved lines, cluster two with five improved lines and cluster three with three improved lines (Fig. S7). The highest yielding improved lines were placed in cluster two with average single plant yield of 31.51 g. Improved lines with the lowest number of days to 50% flowering were grouped in the cluster one.

## Discussion

Marker-assisted breeding of two or more linked and unlinked genes into a single breeding line/variety to achieve the desired trait is termed as “gene introgression/pyramiding”^27^. The disease breeding program involves two necessary steps viz., gene introgression/pyramiding and gene fixation. In our study, the target genes (*Rps2* + *Rmd-c*) were introgressed into the single cross populations of CO 3 and JS 335, independently. Later, the fixation of the gene(s) was carried out in the developed cross population (CO 3 × CNS and JS 335 × CNS). The resistance genes (*Rps2* + *Rmd-c*) coupled with ineffective nodulator (*Rj2*) were separated, and the genotypes with disease resistance and effective nodulator (*rj2*) were developed. Gene introgression can be achieved through genotypic and phenotypic selection. As hypothesized, the trait with a high degree of heritability is much easier to pyramid in fewer generations, while the low heritability trait needs a few more generation^28^.

The conventional breeding procedure is to select the resistant genotype based on phenotype appearances from a vast base population with high favorite allele frequencies. However, gene introgression of multiple traits through conventional breeding is a time-consuming process. Fortunately, the availability of functional marker (*Rps2* + *rj2* + *Rmd-c*: 91F11L marker) linked to the target genes allowed us to introgress the genes quickly, and more efficiently with a minimum level of linkage drag on the target chromosome. In marker-assisted selection (MAS), the availability of robust molecular markers has many selective advantages over the phenotypic plant selection. Firstly, the genotypic selection has made in the two-mapping population (CO 3 × CNS; JS 335 × CNS) at the earlier stage of the plant-development, *i.e*., vegetative stage, resulting in an apparent increase in the genotyping efficiency. Secondly, the selection based on genotyping data allowed us to identify the progenies with desired gene combination in Improved CO 3 and Improved JS 335. Third, we observed a negligible level of genotype × environment (G × E) effects driven impact in the genotypic plant selection, unlike phenotypic screening.

We exerted maximum caution while selecting the molecular marker for screening the progenies carrying *Rps2* + *rj2* + *Rmd-c* genes. It is mandatory that the genetic distance between markers and gene of interest should not be very long. If the marker is far away from the gene, there are more chances of recombination between markers and target gene in each generation^28–30^. In the above condition, the genetic linkage between gene and the markers is greatly affected. The molecular marker, which was used in our introgression program is a functional marker and is within the coding sequence of gene region and had very tight linkage with the donor parent’s R-gene. This marker is expected to enhance the reliability of selection based on genotype, as it helps in direct selection of gene (s) involved in *Phytophthora* rot and powdery mildew resistance.

Meanwhile, we have maintained at least a minimum size of the population to yield a plant carrying all the target allele(s). The frequency and probability of getting the desired plant mainly depend on the number of genes being used in the crossing programme, genetic linkage, and the method/nature of the crossing program. The stringent phenotypic selection in combination with genotyping have yielded success in the introgression of R genes and the effective nodulating gene in the target lines as observed in previous studies^31–35^. Besides, we have selected the genes that are providing race-nonspecific resistance to both *Phytophthora* and powdery mildew pathogens. Resistance build-up to *Phytophthora* rot or powdery mildew was accelerated through functional markers of the *Rps2* and *Rmd-c* genes. Combining multiple resistance genes or QTLs for broad-spectrum resistance to various diseases will also guarantees the durability of resistance. For instance, Cithrameenal et al.^35^ pyramided three BB (*xa5* + *xa13* + *Xa21*) resistance genes for broad-spectrum resistance to BB along with *OsPSTOL* 1 gene in rice.. Hittalmani et al.^36^ and Castro et al.^37^ combined genes originating from three different parents for rice blast and stripe rust in barley, respectively. Successful application of maker assisted selection depends on several factors including the type of marker, strength of linkage, inheritance of trait of interest etc. In recent years, the SNP markers are also becoming extremely popular in marker assisted breeding due to their genome-wide abundance and amenability for high- to ultra-high-throughput detection platforms^38^. Though SNP genotyping is an attractive strategy for background selection, the availability of Haplotype-based SNP marker is a current limitation. Development of haplotype-based SNP markers would greatly facilitate the foreground selection in gene pyramiding/introgression.

To conclude, we have successfully improved the two Indian soybean varieties with enhanced resistance to *Phytophthora* rot and powdery mildew diseases and also achieved effective nodulation in plants. The soybean production in the recent years has been grappling with a host of challenges particularly from diseases, which is prevalent in most of the cropped countries. The best way of overcoming the yield loss is to improve the high-yielding varieties with broad-spectrum disease resistance. From the previous and current research findings, it is evident that the main and perhaps the only long-term solution is to employ the marker-assisted selection in conventional breeding techniques to achieve trait improvement in high yielding varieties. From the current study, three agronomically improved lines were identified with resistance to *Phytophthora* rot and powdery mildew diseases with effective nodulation. The identified superior homozygous near-isogenic lines are being further propagated and carried forward towards large-scale field studies.

## Supplementary information

Supplementary file1
