# Supplementary material for: Improved host-plant resistance to Phytophthora rot and powdery mildew in soybean (Glycine max (L.) Merr.)
Source: Sci Rep. 2020 Aug 18;10:13928. doi: 10.1038/s41598-020-70702-x (PMC7434881; doi:10.1038/s41598-020-70702-x)
Supplement: Supplementary file 1 — Supplementary file1 [file 41598_2020_70702_MOESM1_ESM.doc]

**Improved host-plant resistance to *Phytophthora* rot and Powdery mildew in Soybean (*Glycine max* (L.) Merr.)**

**Jegadeesan Ramalingam^1,2*^, Ganesh Alagarasan^1^, Palanisamy Savitha^1^, Kelsey Lydia^1^ Govindan Pothiraj^1^, Eswaramoorthy Vijayakumar^3^, Rajaprakasam Sudhagar^4^, Amar Singh^5^, Kumari Vedna^6^ and Chockalingam Vanniarajan^3^**

^1^Centre for Plant Molecular Biology and Biotechnology, Tamil Nadu Agricultural University, Coimbatore, ^2^Department of Biotechnology, Agricultural College and Research Institute, Tamil Nadu Agricultural University, Madurai, ^3^Department of Plant Breeding and Genetics, Agricultural College and Research Institute, Tamil Nadu Agricultural University, Madurai, ^4^Department of Pulses, Centre for Plant Breeding and Genetics, Tamil Nadu Agricultural University, Coimbatore - India. ^5^Department of Plant Pathology, Chaudhary Sarwan Kumar Himachal Pradesh Krishi Vishvavidyalaya, Palampur-India. ^6^Department of Plant Breeding and Genetics, Chaudhary Sarwan Kumar Himachal Pradesh Krishi Vishvavidyalaya, Palampur-India.

***Corresponding author**

**Jegadeesan Ramalingam**

Department of Biotechnology,

Agricultural College and Research Institute, Tamil Nadu Agricultural University, Madurai. India.

**Email:** [**ramalingam.j@tnau.ac.in**](mailto:ramalingam.j@tnau.ac.in)

**Supplementary Table S1. Composition of oatmeal agar media and V8 agar media**

| **Oat meal agar media compsition** | **V8 agar media compsition** |
| --- | --- |
| Oat meal (Hi media) - 60g/l  Agar - 12.5g/l  Final pH (at 25 °C) - 7.2±0.2 | V8 juice (Himedia) - 8.3g/l  L-Asparagine - 10g/l  Yeast extract - 2g/l  Calcium carbonate - 2g/l  Glucose - 2g/l  Agar - 20g/l  Final pH (at 25 °C) - 5.7±0.2 |

**Supplementary Table S2. Composition of Yeast mannitol liquid broth**

| **Yeast mannitol liquid broth** | |
| --- | --- |
| Yeast extract (Hi media)  Mannitol  Dipotassium phosphate  Magnesium phosphate  Sodium chloride  Calcium carbonate  Final pH (at 25 °C) | 1g/l  10g/l  0.5g/l  0.2g/l  0.1g/l  1g/l  6.8±0.2 |

**Supplementary Table S3. Polymorphic markers used in two BC_1_F_1_ lines and banding pattern of background marker alleles**

| **S. No** | **Marker** | **Chromo-some** | **CO 3/**  **JS 335 (Recurrent)** | **CNS (Donor)** | **BC_1_F_1_ plant*** | **BC_1_F_1_ plant *** |
| --- | --- | --- | --- | --- | --- | --- |
| 1. | SATT 184 | 1 | AA | BB | AA | AA |
| 2. | SATT 141 | 2 | AA | BB | AB | AB |
| 3. | SATT 257 | 3 | AA | BB | AB | AB |
| 4. | SATT 009 | 3 | AA | BB | AA | AA |
| 5. | SATT194 | 4 | AA | BB | AA | AA |
| 6. | SATT 200 | 5 | AA | BB | AA | AA |
| 7. | SATT545 | 5 | AA | BB | AA | AA |
| 8 | SATT 357 | 6 | AA | BB | AB | AA |
| 9 | SATT 281 | 6 | AA | BB | AB | AB |
| 10 | SATT 307 | 6 | AA | BB | AB | AA |
| 11 | SATT175 | 7 | AA | BB | AA | AA |
| 12 | SATT308 | 7 | AA | BB | AA | AA |
| 13 | SATT 233 | 8 | AA | BB | AA | AA |
| 14 | SATT 409 | 8 | AA | BB | AA | AA |
| 15 | SATT 119 | 8 | AA | BB | AB | AB |
| 16 | SATT 441 | 9 | AA | BB | AA | AA |
| 17 | SATT 046 | 9 | AA | BB | AA | AA |
| 18 | SATT 243 | 10 | AA | BB | AA | AA |
| 19 | SATT 358 | 10 | AA | BB | AA | AA |
| 20 | SATT 509 | 11 | AA | BB | AA | AA |
| 21 | SATT 453 | 11 | AA | BB | AB | AB |
| 22 | SATT 353 | 12 | AA | BB | AA | AB |
| 23 | SATT 302 | 12 | AA | BB | AA | AA |
| 24 | SATT 160 | 13 | AA | BB | AA | AA |
| 25 | SATT 144 | 13 | AA | BB | AA | AA |
| 26 | SATT 063 | 14 | AA | BB | AA | AA |
| 27 | SATT 534 | 14 | AA | BB | AA | AA |
| 28 | SATT 020 | 14 | AA | BB | AA | AA |
| 29 | SATT 231 | 15 | AA | BB | AA | AA |
| 30 | SATT 431 | 16 | AA | BB | AB | AA |
| 31 | SATT 547 | 16 | AA | BB | AA | AA |
| 32 | SATT 226 | 17 | AA | BB | AB | AA |
| 33 | SATT 574 | 17 | AA | BB | AA | AA |
| 34 | SATT 288 | 18 | AA | BB | AA | AA |
| 35 | SATT688 | 18 | AA | BB | AA | AA |
| 36 | SATT 495 | 19 | AA | BB | AA | AA |
| 37 | SATT 373 | 19 | AA | BB | AA | AA |
| 38 | SATT 127 | 20 | AA | BB | AB | AA |
| 39 | SATT 354 | 20 | AA | BB | AA | AA |

* Confirmed true BC1F1 plants from CO3 x CNS combination in foreground analysis

**Supplementary Table S4. Analysis of Variance for agronomic performance of pyramided lines**

| **Source of Variation** | ***SS*** | ***df*** | ***MS*** | ***F*** | ***P-value*** | ***F crit*** |
| --- | --- | --- | --- | --- | --- | --- |
| Rows | 7611.13 | 14 | 543.65 | 3.46 | 0.00 | 1.78* |
| Columns | 168570.8 | 8 | 21071.35 | 134.26 | 0.00 | 2.02* |
| Error | 17577.29 | 112 | 156.94 |  |  |  |
| Total | 193759.3 | 134 |  |  |  |  |

*significance at 5 % level of significance.


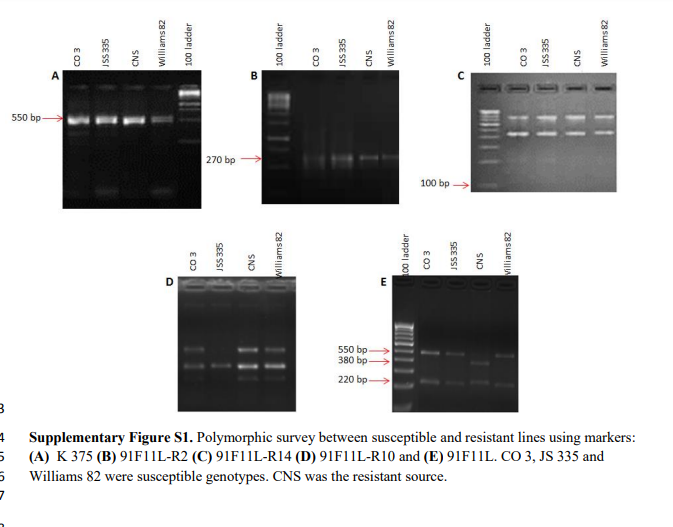


**
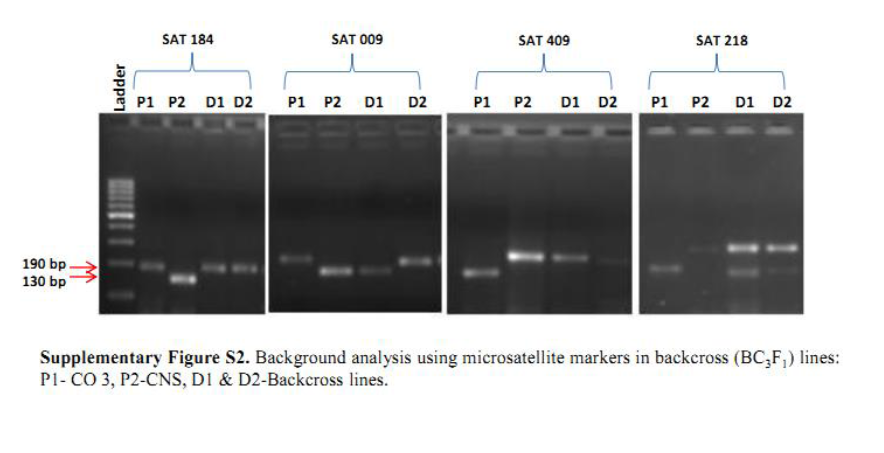
**


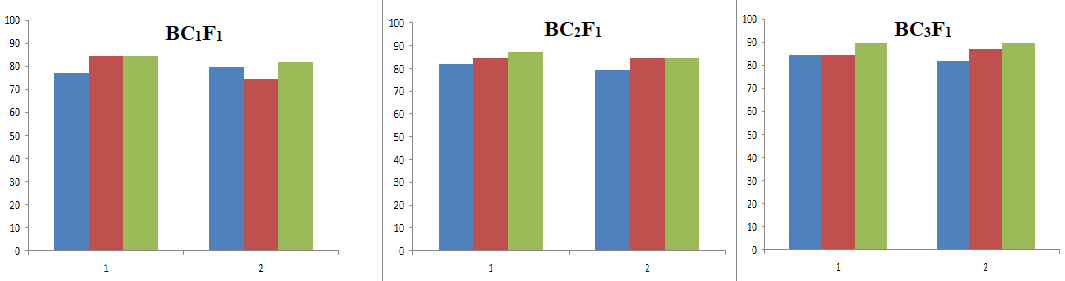


**Supplementary Figure S3.** Recurrent genome contribution in backcross plants of CO 3 × CNS (1) and JS335 × CNS (2) combinations. Blue indicates selected backcross plant 1, maroon indicates selected backcross plant 2 and light green indicates selected backcross plant 3 in two crosses respectively.


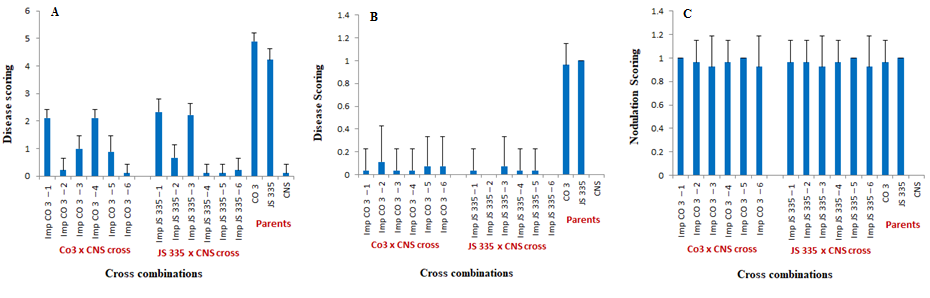


**Supplementary Figure S4.** Distribution of parents (CO 3 and JS 335) and improved genotypes (improved CO 3 and JS 335) means for disease response. (A) Powdery mildew (B) *Phytophthora* and (C) Nodulation efficiency. CNS is the check parents.


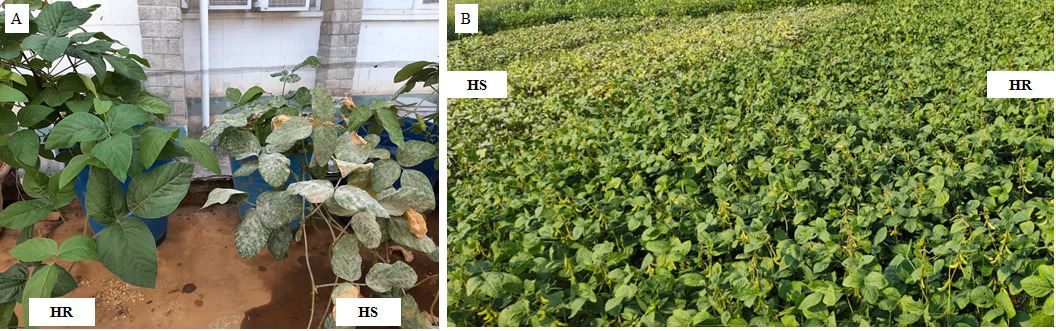


**Supplementary Figure S5.** Whole plant assay (A) and field view (B) of CO 3 (HS) and improved CO 3 (HR) against powdery mildew response. HS indicates highly susceptible. HR indicates highly resistant.


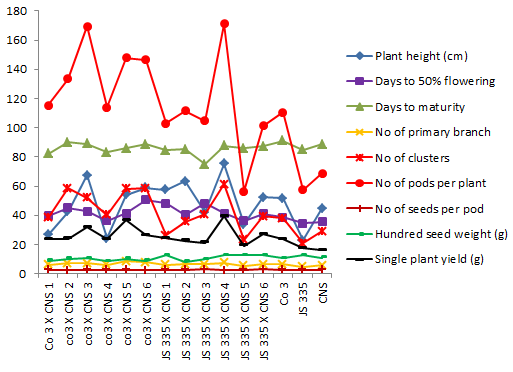


**Supplementary Figure S6.** Agronomic performance of improved soybean lines for different biometrical traits. CO 3 and JS 335 are popular soybean varieties and CNS is the donor parent.

**
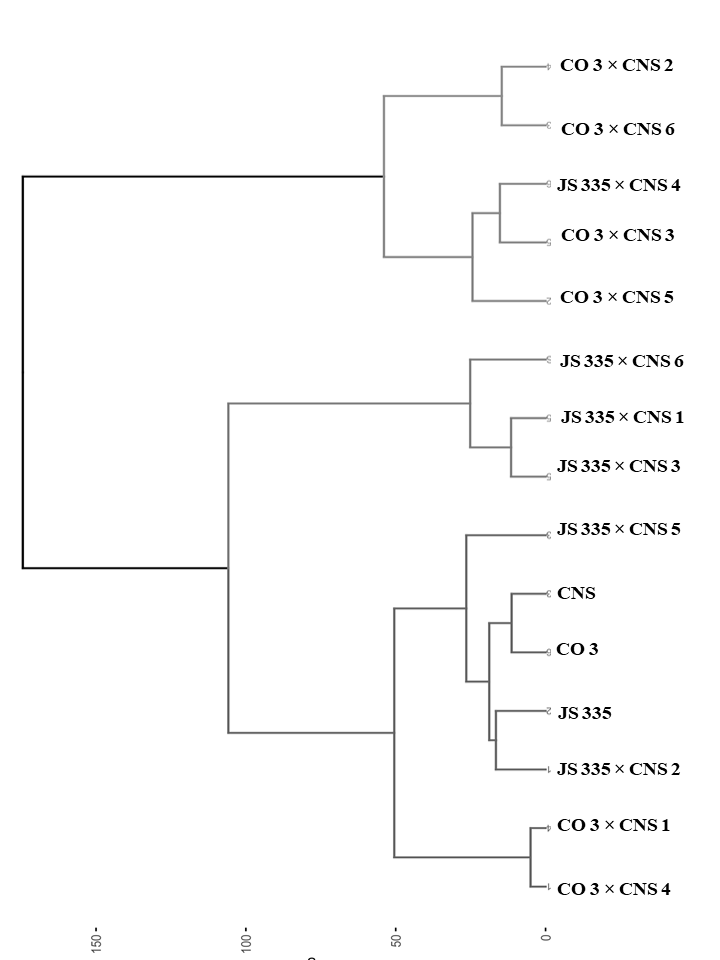
**

**Supplementary Figure S7.** Agglomerative clustering method based on nine quantitative characters in pyramided lines
